# Bilateral Spontaneous Pneumothoraces in a Case of Tricuspid Valve Endocarditis and Septic Emboli: A Rare Complication

**DOI:** 10.1155/2024/3049691

**Published:** 2024-05-21

**Authors:** Nim Chan, Bryan Dunn

**Affiliations:** East Carolina University, Department of Pulmonary and Critical Care Medicine, Greenville, USA

## Abstract

Acute hypoxemic respiratory failure from infective endocarditis with septic emboli has been attributed to the vicious cycle of tissue damage and inflammatory cytokine response. Spontaneous pneumothorax is a rare complication and can be a late-onset presentation despite appropriate antibiotic therapy. We present a rare case of bilateral spontaneous pneumothoraces in a patient with tricuspid valve endocarditis and septic pulmonary emboli. We suspect that the profound inflammatory response from two different bacterial pathogens and the peripheral location of the septic thrombosis are the basis of the development of bilateral pneumothorax development in our patient.

## 1. Introduction

Septic pulmonary emboliis a known complication of right-sided infective endocarditis and is most commonly associated with tricuspid valve vegetations. Spontaneous pneumothorax secondary to septic emboli, however, is rare and unusual with less than a dozen cases documented in literature [1]. We present a case of bacteremia with tricuspid valve infective endocarditis complicated by recurrent bilateral pneumothoraces and parapneumonic effusions from progressive necrotizing septic emboli without underlying bullous disease [1–4].

## 2. Case

This is a case of a 46-year-old male with a history of uncontrolled type II diabetes, left above knee amputation, and opiate abuse who was presented to the emergency department with shortness of breath and fatigue for two days. He was found to be in vasopressor-dependent shock and acute hypoxemic respiratory failure requiring endotracheal intubation and mechanical ventilation. Initial vitals were temperature of 40.3°C, blood pressure of 83/53 mmHg, and tachycardia of 137 per minute with respiratory rate of 41 per minute. Blood work was significant for leukocytosis of 48 × 10^3^ cells/*μ*L (neutrophil predominant), lactic acid of 8.3 mmol/L, LDH of 698 U/L, total bilirubin of 11 mg/dL, direct bilirubin of 7.7 mg/dL, and alkaline phosphatase of 272 U/L.

**Figure 1:**
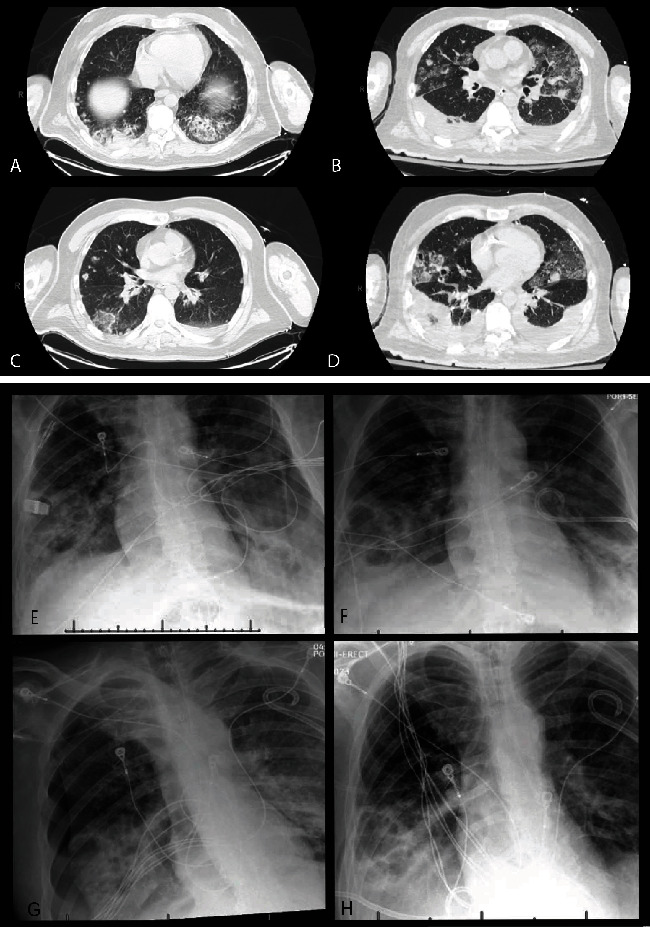
(A, B) Initial CT chest images showing bilateral nodular lesions consistent with embolic phenomenon along with basilar ground glass opacities. (C, D) Repeat CT chest images showing progression of nodular embolic lesions into cavitary lesions. (E, F) Right pneumothorax—pre- and postchest tube placement. (G, H) Left pneumothorax—pre- and postchest tube placement.

Empiric antibiotic treatment was initiated with meropenem and vancomycin for presumed pneumonia as well as possible acute cholecystitis due to the elevated bilirubin. Initial blood and sputum cultures resulted in methicillin-sensitive *Staphylococcus aureus* (MSSA), and antibiotics were narrowed to IV cefazolin. A large tricuspid valve vegetation was visualised on transesophageal echocardiogram, and computed tomography (CT) of the chest reported numerous bilateral pulmonary nodules, some of which appeared cavitary (Figures 1(A), 1(B), 1(C), and 1(D)). These findings were consistent with metastatic MSSA infection. There was no evidence of baseline chronic pulmonary disease on imaging. The patient hospital course was complicated by the development of spontaneous pneumothorax of first, the right lung (Figures 1(E) and 1(F)), followed two days later pneumothorax of the left lung (Figures 1(G) and 1(H)). Cardiothoracic surgery was consulted for this patient. He was found not to be a candidate for surgery due to hemodynamic instability, nor was he a candidate for aspiration thrombectomy due to the size of the tricuspid valve vegetation. A repeat bronchoalveolar lavage 5 days after admission showed new growth of *Pseudomonas aeruginosa*, and meropenem was reintroduced for treatment. Given his worsening clinical status, the family opted to proceed with comfort care.

## 3. Discussion

The mechanism of spontaneous pneumothorax can be separated into three categories: (1) mechanical, (2) structural, and (3) necrotizing. Mechanical-induced pneumothorax is related to direct trauma to the thoraces or barotrauma with high peak pressure from mechanical ventilation. Structural changes include multiple lung conditions including bullous diseases (lymphangioleiomyomatosis, Birt-Hogg-Dubé syndrome, or *Pneumocystis jirovecii* pneumonia), cancer, and connective tissue disease such as Marfan's or Ehlers-Danlos as well as rheumatologic diseases [3, 5].

Our patient had septic emboli which is rarely associated with pneumothoraces.

He had neither mechanical nor structural factors; while being maintained on mechanical ventilation, his ventilator settings were adjusted to prevent excessive positive pressure. Chest CT from prior hospitalizations and current admission did not show any obvious chronic bullous or structural lesions. This suggests that the mechanism of this patient developing pneumothorax is most likely necrotic and destructive in nature.

We theorize that this patients' pneumothoraces developed as a consequence of both the peripheral location of the septic emboli and the profound inflammation induced by two different bacterial infections (MSSA and *P. aeruginosa*). His elevated bilirubin was felt to be secondary to distributive shock from the pneumonia rather than true acute cholecystitis, and therefore, no surgical intervention was warranted. There are currently seven reported cases in literature of septic pulmonary emboli with pneumothorax with our case being the eighth. All cases have shown *Staphylococcus aureus* as the pathogen causing the endocarditis [6]. Further necrosis secondary to the *P. aeruginosa* infection that developed in addition to the MSSA cavitary lesions may have increased his risk of destruction of the pulmonary parenchyma resulting in his pneumothoraces.

## 4. Conclusion

Pneumothoraces are a rare complication of septic pulmonary emboli. In cases of cavitary formation, spontaneous pneumothorax should be in the differential in patients who develop worsening respiratory symptoms despite appropriate antibiotic treatment.
